# Detection of Invasive Mosquito Vectors Using Environmental DNA (eDNA) from Water Samples

**DOI:** 10.1371/journal.pone.0162493

**Published:** 2016-09-14

**Authors:** Judith Schneider, Alice Valentini, Tony Dejean, Fabrizio Montarsi, Pierre Taberlet, Olivier Glaizot, Luca Fumagalli

**Affiliations:** 1 Laboratory for Conservation Biology, Department of Ecology and Evolution, University of Lausanne, Biophore, CH-1015, Lausanne, Switzerland; 2 SPYGEN, Savoie Technolac, 73370, Le Bourget du Lac, France; 3 Istituto Zooprofilattico Sperimentale delle Venezie, Viale dell’Università 10, 35020, Legnaro, Padova, Italy; 4 Université Grenoble-Alpes, Laboratoire d’Ecologie Alpine (LECA), F-38000, Grenoble, France; 5 Centre National de la Recherche Scientifique, Laboratoire d'Ecologie Alpine (LECA), F-38000, Grenoble, France; 6 Museum of Zoology, Place de la Riponne 6, CH-1014, Lausanne, Switzerland; 7 Department of Ecology and Evolution, University of Lausanne, Biophore, CH-1015, Lausanne, Switzerland; University of Hyogo, JAPAN

## Abstract

Repeated introductions and spread of invasive mosquito species (IMS) have been recorded on a large scale these last decades worldwide. In this context, members of the mosquito genus *Aedes* can present serious risks to public health as they have or may develop vector competence for various viral diseases. While the Tiger mosquito (*Aedes albopictus*) is a well-known vector for e.g. dengue and chikungunya viruses, the Asian bush mosquito (*Ae*. *j*. *japonicus*) and *Ae*. *koreicus* have shown vector competence in the field and the laboratory for a number of viruses including dengue, West Nile fever and Japanese encephalitis. Early detection and identification is therefore crucial for successful eradication or control strategies. Traditional specific identification and monitoring of different and/or cryptic life stages of the invasive *Aedes* species based on morphological grounds may lead to misidentifications, and are problematic when extensive surveillance is needed. In this study, we developed, tested and applied an environmental DNA (eDNA) approach for the detection of three IMS, based on water samples collected in the field in several European countries. We compared real-time quantitative PCR (qPCR) assays specific for these three species and an eDNA metabarcoding approach with traditional sampling, and discussed the advantages and limitations of these methods. Detection probabilities for eDNA-based approaches were in most of the specific comparisons higher than for traditional survey and the results were congruent between both molecular methods, confirming the reliability and efficiency of alternative eDNA-based techniques for the early and unambiguous detection and surveillance of invasive mosquito vectors. The ease of water sampling procedures in the eDNA approach tested here allows the development of large-scale monitoring and surveillance programs of IMS, especially using citizen science projects.

## Introduction

The globalisation of trade and travel has boosted biological invasions of animal and plant species by facilitating their worldwide dispersal, often causing detrimental environmental, economical or sanitary impacts [1, 2]. Invaders can alter the dynamics and functioning of a whole ecosystem, like the New Zealand mudsnail *Potamopyrgus antipodarum* [3]. Economical charges caused by invasive species are illustrated for example by the recent effects of the spotted-wing drosophila (*Drosophila suzukii*) on the fruit-producing sector in Southern Europe (e.g. [4]). Invasive mosquito species (IMS) are of particular concern for human and animal health since they are confirmed or potential vectors for several pathogens [5]. As a consequence, since the outbreaks in Italy in 2007 of chikungunya fever transmitted by the invasive Tiger mosquito *Aedes albopictus*, awareness has risen for at-risk areas for new or re-emerging mosquito-borne diseases in Europe [6, 7]. Similarly, Zika virus has recently spread vectored by the invasive *Aedes aegypti* mosquito across the Pacific Ocean to the New World, where the infection has reached pandemic levels [8]. At least five exotic *Aedes* mosquito species with different degrees of public health impact are established in Europe (reviewed in [9]). Preventing the dispersal and establishment of the invaders is often easier and less costly than their eradication. Early detection is thus crucial for the success of management actions [10], but efforts can be tremendously high in case of low-level presence of an organism that is difficult to detect [11].

This study investigates a new approach to monitor three IMS belonging to the genus *Aedes* (Diptera: Culicidae), based on the analysis of environmental DNA (eDNA) from water samples collected in the field. The Tiger mosquito (*Aedes* (*Stegomyia*) *albopictus* (Skuse 1894)) originates in South East Asia and has begun its worldwide expansion in the late 1970s. Nowadays it is present in many regions of the Americas, Africa and Southern Europe (reviewed in [12]). The native range of the Asian bush or Asian rock pool mosquito (*Aedes* (*Finlaya*) *japonicus japonicus* (Theobald 1901)) is also East Asia and Eastern Russia. Today the species is established in the USA and several central European countries (reviewed in [13]). *Aedes* (*Finlaya*) *koreicus* (Edwards, 1917) is phylogenetically close to *Ae*. *j*. *japonicus* and originates from the same region. The first finding in Europe dates back to 2008 [14]. Since 2011 it is locally established in Italy [15, 16] and was recently detected in Switzerland and southern Germany [17, 18].

Members of the *Aedes* genus are known vectors for numerous viral diseases. Among the studied IMS, *Ae*. *albopictus* causes the most sanitary concerns as it is a vector for several viruses, notably dengue and chikungunya, and has been shown to be a competent vector for Zika virus both experimentally and in the field [19, 20]. It is at the origin of the chikungunya fever outbreaks in 2007 in Italy [21], the dengue fever in Croatia in 2010 [9], and of both viruses in France the same year [22]. Vector competences have been documented for *Ae*. *j*. *japonicus*, which has been found infected in the field and in the laboratory for a number of viruses, including dengue, chikungunya, West Nile and Japanese encephalitis [7, 23, 24]. *Aedes koreicus* has also been found infected in the field with the Japanese encephalitis virus, so far without prove of transmission [9].

IMS benefit from both climate change and globalised trade and traffic. The principal introduction mode of container breeding IMS is the transport of dry-resistant eggs through the used tire trade [9, 25]. Species distribution models combining environmental and land cover variables under future climate change scenarios predict further spread, e.g. for *Ae*. *albopictus* and *Ae*. *koreicus* in Europe and worldwide [26–29].

Given their medical importance and projected future dispersal, the early detection of IMS and intensive surveillance of large areas is crucial. Traditional monitoring methods based on the morphological identification of different life stages may be problematic for the invasive *Aedes* species [30]. Another method used for specific identifications of e.g. *Aedes* eggs is matrix assisted laser-desorption/ionization time of flight mass spectrometry (MALDI TOF MS; [31]), for which sampled eggs must be individually prepared before analysis. DNA-barcoding with conventional polymerase chain reaction (PCR) and subsequent sequencing has also been used to confirm morphological determinations of single mosquito specimens [32, 33] and for invasive *Aedes* in particular [14, 34], but this is not the method of choice for large-scale surveillance in terms of time and money. A faster option to determine unknown mosquito specimens is the use of species-specific quantitative real-time PCR (qPCR), which has been applied to the identification of individual specimens [30, 35].

In recent years, there has been an increased interest in environmental DNA (eDNA)-based methods for a broad array of environments and research fields due to technical advances (reviewed e.g. in [36–39]). All organisms leave traces of DNA in their environment through faeces, urine, hair, skin or cells, which can be subsequently analysed. Ficetola *et al*. [40] were the first to use eDNA for the detection of an invasive species in an aquatic environment, an approach which has since been implemented in several other studies (e.g. [3, 11, 41–44]). Although technically challenging, eDNA can be especially useful for species with a low detection probability, problematic morphological identification and cryptic life stages such as IMS. However, this approach has so far never been applied to mosquito species in general or to IMS in particular.

In this study, we developed and tested for the first time an eDNA approach for the detection and identification of IMS using water samples collected in the field from seven European countries. We compared qPCR TaqMan assays specific for three IMS (*Aedes albopictus*, *Ae*. *j*. *japonicus*, *Ae*. *koreicus*) and a DNA-metabarcoding approach with traditional survey based on larval sampling. Our objective was to determine if eDNA analysis from water samples performed better than traditional survey methods and could be used to reliably identify IMS and implement large-scale monitoring and surveillance programs.

## Methods

### Ethics statement

No permits were required for sampling at any of the sites in this study. Field sampling did not involve any endangered or protected species.

### Sample collection

**Fig 1:**
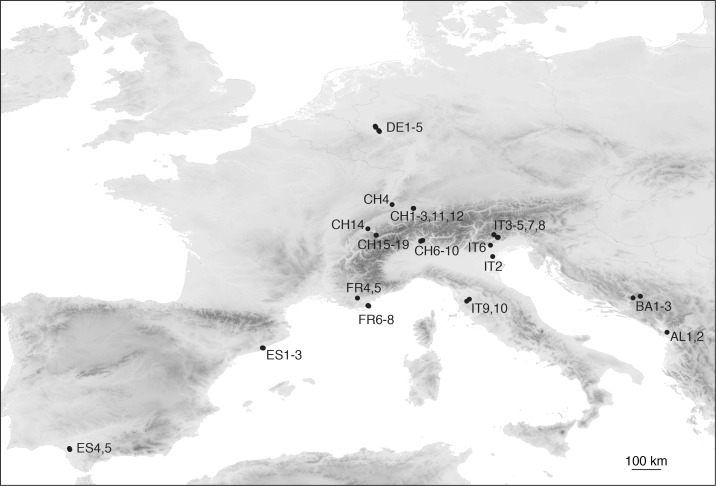
Map showing all sampling locations across Europe. AL: Albania; BA: Bosnia-Herzegovina; CH: Switzerland; DE: Germany; ES: Spain; FR: France; IT: Italy. Location details are given in S1 Table.

The target IMS were *Aedes albopictus* (Tiger mosquito), *Ae*. *j*. *japonicus* (Asian bush mosquito) and *Ae*. *koreicus*. Water samples originated from 46 natural water bodies sampled from June to September 2015 in seven European countries (Fig 1 and S1 Table). This period corresponds to summer season in temperate latitudes, characterized on average by low precipitation and high temperature. A first group comprised 30 samples where the presence of the target IMS in the water was confirmed at the time of sampling by morphological identification of larvae. The sampling and identification of mosquito larvae (traditional survey thereafter) was carried out by expert entomologists following the methods reported in [16, 45]. A second set of 16 water samples was collected at suitable breeding sites (i.e. within the known or potential geographical range of the species; [46]), but with no direct observation of the IMS during the sampling procedure. Sampling kits were prepared in the laboratory under a laminar flow hood. Each kit included 4 x 50 mL Falcon tubes with 33 mL 100% ethanol (EtOH), gloves and sterile dippers. Water samples were collected following the protocol of Ficetola *et al*. [40] with some modifications. At each sampling site, 3 x 15 mL water was taken and added to the Falcon tubes. One negative control was collected at each site to monitor the quality of the kit and possible contaminations across sites, by adding 15 mL of bottled water to the remnant Falcon tube. Water samples were stored at 4°C prior to DNA extraction.

We used larvae and/or adult tissues of target (the three studied IMS) and non-target species (14 additional mosquito species) as starting material for preliminary setup and for cross-amplification tests (S2 Table).

### DNA extraction

DNA extractions from water samples were performed using the DNeasy Kit following the manufacturers‘ instructions with some modifications. Prior to DNA extraction, 1.5 mL sodium acetate (NaOAc) 3M *pH* 5.2 was added to each 50 mL Falcon tube before being incubated at -20°C overnight [40]. The Falcon tubes were then centrifuged at 6°C for 30 min at 8,000 rpm. The supernatant was discarded and 20 mL EtOH added before another centrifugation at 6°C for 10 min. The supernatant was once more discarded and the tubes kept at 65°C for 10 min to evaporate the remaining EtOH. The pellets were dissolved in 720μL ATL buffer and 80 μL proteinase K, transferred to 2 mL Eppendorf tubes and incubated overnight at 56°C and 50 rpm. All further steps were performed according to the DNeasy Blood and Tissue protocol. All three subsamples taken at one site were passed on the same DNeasy column. After the first application of elution buffer to the column, an additional 30 s spin at 100 rpm was carried out to obtain higher DNA yields, before spinning at 13,200 rpm for 1 min. The eDNA was eluted in 300 μL buffer. Extraction blanks were included in each DNA extraction session to control for possible contaminations. Extracted DNA was quantified using a QuBit® Fluorometer 2.0 (Life Technologies, Carlsbad, USA) following the recommended protocol, then stored at -20°C. Samples were extracted within one month after collection. DNA extractions, as well as preparation of sampling kits, reagent dilutions and pre-PCR mixtures, were carried out in a separate laboratory dedicated to pre-PCR operations.

Adults and larvae were decapitated prior to extraction in order to diminish potential inhibition [47] and cuts were made along the abdomen to allow for complete lysis. DNA extractions were performed using the DNeasy Blood and Tissue Kit (Qiagen GmbH, Hilden, Germany) following the manufacturers‘ instructions. In the final step, DNA was eluted in 100 μL elution buffer and stored at—20°C.

### Real-time quantitative PCR (qPCR)

Primers and probe for *Ae*. *albopictus* were developed by Hill *et al*. [35] and optimized by Van de Vossenberg *et al*. [30]. They amplify 35 base-pairs (bp) of the ribosomal internal transcribed spacer 1 (ITS 1). For *Ae*. *j*. *japonicus*, primers and probe were developed by the latter authors, amplifying a 77 bp fragment of the mitochondrial DNA (mtDNA) cytochrome oxidase subunit I (COI). We newly designed primers and probe for *Ae*. *koreicus* amplifying a 77 bp COI fragment, by aligning sequences of *Ae*. *albopictus* (GenBank accession nr. KM613121), *Ae*. *j*. *japonicus* (KP076256) and *Ae*. *koreicus* (KM457600). All primers and probes were then validated *in silico* and *in vitro*.

The *in silico* analysis was performed with an electronic PCR using ecoPCR software [48] on the EMBL-Bank release 127 (April 2016). In order to test for potential cross-amplifications, qPCRs were carried out in technical duplicates with all primers and probe sets on the three studied IMS and on 14 non-target species potentially co-occurring in the same geographical range (S2 Table). In particular, we repeated the analysis for several species already tested for cross-amplification by Van de Vossenberg *et al*. [30] to confirm that the cross-amplification tests were not dependent on the different qPCR cycle number between studies. Ten-fold dilution series of genomic DNA extracted from tissue specimens ranging from 10 ng x μL^-1^ to 0.01 fg x μL^-1^ were run in triplicate to determine the assays‘ sensitivities (the level of detection LOD, i.e. the minimum quantity of target DNA detectable in the sample, and the level of quantification LOQ, i.e. the lowest level of target DNA that yields an acceptable level of precision and accuracy).

The amplification of tissue DNA and eDNA was performed using an Applied Biosystems 7500 Real-time PCR system (Life Technologies). The thermal profile was the following: 2 min at 50°C, 10 min at 95°C, 55 cycles of 15 s at 95°C and 60 s at 64°C for *Ae*. *albopictus*, and at 60°C for *Ae*. *j*. *japonicus* and for *Ae*. *koreicus*. For the *Ae*. *albopictus* assay, each TaqMan reaction (TaqMan Gene Expression Master Mix; Life Technologies) contained 300 nM of each primer and the dual-labelled hydrolysis probe (with 6-carboxy-fluorescein (FAM) as a reporter at the 5' end and Black Hole Quencher Amidites (BHQ1) at the 3' end), while 450 nM of each primer and 300 nM of the probe for *Ae*. *j*. *japonicus* (slightly modified from the conditions in Van de Vossenberg *et al*. [30]). For the new *Ae*. *koreicus* assay, each reaction contained 300 nM of the forward primer (Akore-f: 5'-CCCAGATATAGCCTTCCCCCG-3'), 450 nM of the reverse primer (Akore-r: 5'-GGATAAACAGTTCATCCTGTCCCAG-3') and 300 nM of the probe (FAM-Akore-probe-BHQ1: 5'-CTCCCTCATTAACTCTACTACTTTCAAGAAGTATAGTAG-3'). The final volume of each reaction was 20 μL, containing 2 μL of DNA extraction. To ensure high levels of detection and increase confidence in the results, each sample was tested in 12 technical replicates [49]. Negative controls as well as three positive standards in triplicate were included in all qPCR assays. The positive standards consisted of non diluted target DNA, a dilution of target DNA (10 pg x μL^-1^) and a positive eDNA sample. For *Ae*. *koreicus*, two additional dilutions were added (0.1 ng x μL^-1^, 0.1 pg x μL^-1^).

All 46 water samples collected in the field were analyzed by qPCR. According to the confirmed or potential occurrence (i.e. direct observation during sampling or known geographical range) of each species in each sampling location, overall 31 samples were tested for the presence of *Ae*. *albopictus*, 17 for *Ae*. *j*. *japonicus* and 14 for *Ae*. *koreicus*. All samples showing less than 12 amplified replicates were tested for inhibition. For this purpose, each sample was spiked with 1 μL of an external barn owl (*Tyto alba*, Aves) plasmid (pGEMT-ESR1V2; GenBank Accession nr. KX108739) and amplified with the corresponding primers (ESR1_657Fw: 5'- CACCATCGACAAGAACAGAAGA-3' and ESR1_1129Rev: 5'- AACCAGGCACATTCCAGTAGAT-3') and probe (FAM-ESR1_685TQM-BHQ1: 5'- TGCCAGGCTTGCCGACTAAGAAAA-3'). The final volume of 20 μL contained 1 μL plasmid (4.7 pg x μL^-1^) and 1 μL sample solution. The reaction mixture contained 1x TaqMan Gene Expression Master Mix and 150 nM of each primer and the probe. The samples were tested in duplicates, with one duplicate being 10-fold diluted to account for the possibility that this might enhance amplification in case of inhibitors [50]. The qPCR thermal profile was 2 min at 50°C, 10 min at 95°C, 40 cycles of 15 s at 95°C and 60 s at 60°C. A standard curve was established from the plasmid amplification in technical duplicate.

In order to investigate eDNA persistence in water, larvae of *Ae*. *albopictus* were reared for 12 days at laboratory conditions (24–25°C) in containers filled with 500 mL water at two different densities (10 and 15 larvae, respectively), 3 replicates each. After 12 days (corresponding to day 1 thereafter), all larvae were removed and a sample of 15 mL water was taken from each of the 6 containers, to which we added 33 mL EtOH and 1.5 mL NaOAc 3M *pH* 5.2. Water samples were at days 1, 2, 3, 4, 5, 10, 15 and 19 (one sample at day 15 was discarded for technical reasons). Samples were stored at 4°C prior to DNA extraction, and qPCRs were performed with the same conditions as described above for *Ae*. *albopictus* in 3 technical replicates.

### Next-generation sequencing (NGS)

A subset of 34 samples representative of the species' geographical distribution was amplified with the primers Culicidae (Culicidae-f: 5'-ACGCTGTTATCCCTAAGGTAACTTA-3'; Culicidae-r: 5'-GACGAGAAGACCCTATAGATCTTTAT-3') and sequenced using next-generation sequencing. These primers were first designed with the *ecoprimers* software [51] on a collection of all mitochondrial DNA sequences present in GenBank and manually adjusted using the *ecoPCR* software [48]. They were then validated *in silico* on the EMBL-Bank release 127 (April 2016) for the target taxonomic group Culicidae using *ecoPCR* as described in [52], in order to amplify a 146 bp sequence of the mtDNA 16S rDNA gene. To enable the subsequent assignment of sequences to their respective sample, both the forward and reverse primers were 5‘-labelled with identical tags of eight nucleotides. Tags were designed with the *oligoTag* program included in the *OBITools* package (http://metabarcoding.org/obitools; [53]), with five differences between tags to provide the assignment of reads to samples even in case of sequencing errors [54]. Each tag was preceded by NNN [55].

The amplification mixture contained 1 U of AmpliTaq Gold Polymerase (Applied Biosystems, Foster City, USA), 1x PCR Gold buffer, 2 mM of MgCl_2_, 0.2 mM of each dNTPs, 0.5 μM of each tagged forward and reverse primers and 0.2 mg/mL of bovine serum albumin (BSA, Roche Diagnostics, Basel, Switzerland). The final volume was 25 μL including 3 μL of eDNA extraction. Each sample was amplified in eight replicates with a PCR 2720 Thermal Cycler (Applied Biosystems). The amplifications started with an initial denaturation for 3 min at 95°C, followed by 50 cycles of 30 s at 95°C, 30 s at 60°C and 30 s at 72°C, with a final elongation at 72°C for 5 min. Extraction blanks were included as well as a negative PCR control. The products of the eight replicates were pooled after the PCR and visualised on 1.5% agarose gel stained with ethidium bromide. The amplicons were purified using a MinElute PCR purification kit (Qiagen) with a final elution in 15 μl buffer. Before sequencing, purified DNA was titrated again using capillary electrophoresis. Several purified PCR products were pooled in equal volumes, to achieve an expected sequencing depth of 100,000 reads per DNA sample.

Library preparation and sequencing were performed at Fasteris facilities (Geneva, Switzerland). Libraries were prepared using Metafast protocol (www.fasteris.com/metafast) and a pair-end sequencing (2x125 bp) was carried out using an Illumina Hiseq 2500 sequencer (Illumina, San Diego, CA, USA) with the HiSeq SBS Kit v4 (Illumina, San Diego, CA, USA) following the manufacturer’s instructions. The samples were sequenced in two lanes in two different HiSeq Flow Cell v4.

For the assignment of sequences to taxa, a local sequence reference database was created by blasting the Culicidae primers against the European Nucleotide Archive (ENA) release 123 (May 2015; http://www.ebi.ac.uk/ena) and extracting all sequences with up to three mismatches per primer using ecoPCR software [48] for taxa belonging to family Culicidae. Two specimens of *Ae*. *koreicus* (1 adult, 1 larvae) were analyzed to generate a sequence of the 16S rDNA, which was missing for this species in the local reference database. The amplification mixture was the same as for the metabarcoding amplifications, except that the Culicidae primers were not 5'-tagged. The final volume was 25 μL including 2 μL of DNA solution, the thermal profile being the following: 3 min at 95°C, followed by 35 cycles of 30 s at 95°C, 30 s at 60°C and 30 s at 72°C, and 5 min at 72°C. Purification was performed with the Wizard® SV Gel and PCR Clean-Up System (Promega, Madison, USA) and sequencing made with standard Sanger sequencing at Microsynth facilities (Balgach, Switzerland).

### Data analyses

The threshold for samples to be considered positive or negative in a qPCR replicate assay varies among eDNA studies [56]. We considered a sample positive if at least 2 replicates out of 12 show exponential amplification curves.

The sequence analysis of the metabarcodes obtained after the NGS was done as described in Valentini *et al*. [52], using the *OBITools* package [53]. Filters were applied to exclude erroneous sequences, e.g. all sequences shorter than 20 bp and occurring less than ten times were deleted. For the assignment of sequences to taxa in the local database, a similarity of ≥98% was required.

DNA detectability was tested as a binomial response variable with a linear mixed-effects model using the lmer function in the lme4 package for R [57]. Several models were fitted with initial larval density, days and interactions successively added to the null model. Container was used as a random variable to take into account multiple sampling in a same container. The relative performance of the models was compared with Akaike information criteria [58]. Probabilities of variables of the best model were obtained with a one-way ANOVA.

## Results

The *in silico* analysis validated the specificity of the *Ae*. *albopictus*, *Ae*. *j*. *japonicus* and the newly designed *Ae*. *koreicus* qPCR primers and probe. The *in silico* analysis of the Culicidae primers (S1 Fig) indicated a high taxonomical coverage (i.e. the proportion of species amplified in the target group; 0.9677) and high taxonomical discrimination (i.e. the discrimination capacity at the species, genus and family levels among the amplified taxa; 86%, 100% and 100%, respectively). Analytical *in vitro* specificity of the qPCR assays was confirmed as all three primer pairs and probes were species-specific, amplifying none of the other target and non-target species studied. For the *Ae*. *albopictus* and *Ae*. *j*. *japonicus* assays, real-time amplification efficiency was 96.9% (y = -3.3978x+37.287) and 93.5% (y = -3.4879x+45.236), and coefficient of correlation R^2^ of standard curve was 0.9998 and 0.9956, respectively. For the new *Ae*. *koreicus* assay, amplification efficiency was 97.5% (y = -3.3826x+44.354) and coefficient of correlation R^2^ was 0.9992. The calculated LOD and LOQ for *Ae*. *albopictus* were 0.72 and 2.11 fg x μL^-1^, 11.94 and 34.23 fg x μL^-1^ for *Ae*. *j*. *japonicus* and 14.87 and 43.97 fg x μL^-1^ for *Ae*. *koreicus*, respectively (S2 Fig). No indication of inhibition was found in our tests (qPCR efficiency = 99% (y = -3.344x+39.868); R^2^ = 0.99725), with the exception of a single sample (FR4). This sample showed a delayed amplification for the non-diluted replicate, whereas the 10-fold diluted sample was amplified as expected (confirming that dilution efficiently reduced qPCR inhibition). All field, DNA extraction and amplification negative controls were negative, and all qPCR positive standards were correctly amplified.

Table 1 summarizes the number of detection events using traditional and qPCR surveys (all 46 water bodies). The presence of at least one of the three IMS has been detected in 34 out of 46 (74%) sites whatever the approach used, this number varying from 55 to 82% when looking at the three IMS separately. The corresponding detection probabilities (i.e. the probability that the target IMS is detected given that the target IMS is present at the sampling site) are higher for the qPCR than for the traditional survey, both overall and for the three species separately.

**Table 1:** Number of detection events (*D*.*E*.) and detection probabilities (*D*.*P*.) with traditional survey (larval sampling) and qPCR for the three studied invasive mosquito species (IMS), in the 46 water bodies where both methods have been applied. N: number of sites analyzed.

	N	*D*.*E*. traditional	*D*.*E*. qPCR	No. of sites where the species was detected	*D*.*P*. traditional	*D*.*P*. qPCR
*Ae*. *albopictus*	31	15	16	17	88%	94%
*Ae*. *koreicus*	14	5	8	9	56%	89%
*Ae*. *j*. *japonicus*	17	12	13	14	86%	93%
**OVERALL**	**46**	**30**	**31**	**34**	**88%**	**91%**

Table 2 shows the number of detection events when comparing traditional, qPCR and NGS surveys for the subset of 34 water bodies where the three methods have been applied. Twenty-eight sites out of 34 analyzed (82%) have been found positive for at least one of the three studied IMS whatever the method employed, this value spanning from 62.5 to 79% when looking at the three IMS separately. Detection probabilities are in all comparisons identical or higher for qPCR than for the two other approaches, and higher for NGS than for the traditional approach for *Ae*. *koreicus* and *Ae*. *j*. *japonicus* but not *Ae*. *albopictus*.

**Table 2:** Number of detection events (D.E.) and detection probabilities (D.P.) with traditional survey (larval sampling), qPCR and NGS for the three studied invasive mosquito species (IMS), in the 34 water bodies where the three methods have been applied. N: number of sites analyzed.

	N	*D*.*E*. traditional	*D*.*E*. qPCR	*D*.*E*. NGS	No. of sites where the species was detected	*D*.*P*. traditional	*D*.*P*. qPCR	*D*.*P*. NGS
*Ae*. *albopictus*	24	14	15	12	15	93%	100%	80%
*Ae*. *koreicus*	12	4	8	5	8	50%	100%	63%
*Ae*. *j*. *japonicus*	14	9	10	10	11	82%	91%	91%
**OVERALL**	**34**	**25**	**27**	**24**	**28**	**89%**	**96%**	**86%**

**Fig 2:**
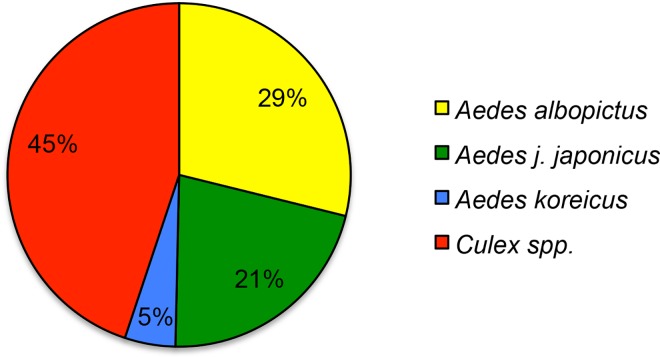
Diagram showing the percentage of assigned Culicidae species after the next-generation sequencing of 34 water samples and bioinformatic sequence analyses applying a 98% similarity threshold.

For the 34 samples analysed with NGS, the total number of reads before applying the filtering parameters was 4,618,312, corresponding on average to 135,833 reads per sample. After applying all the filters, 2,267,458 reads (49.1%, corresponding on average to 66,690 reads per sample) were assigned to 4 different taxa of the Culicidae family (Fig 2 and S3 Table). S1 Table shows the number of positive qPCR replicates and the number of filtered sequences obtained for each sample.

**Fig 3:**
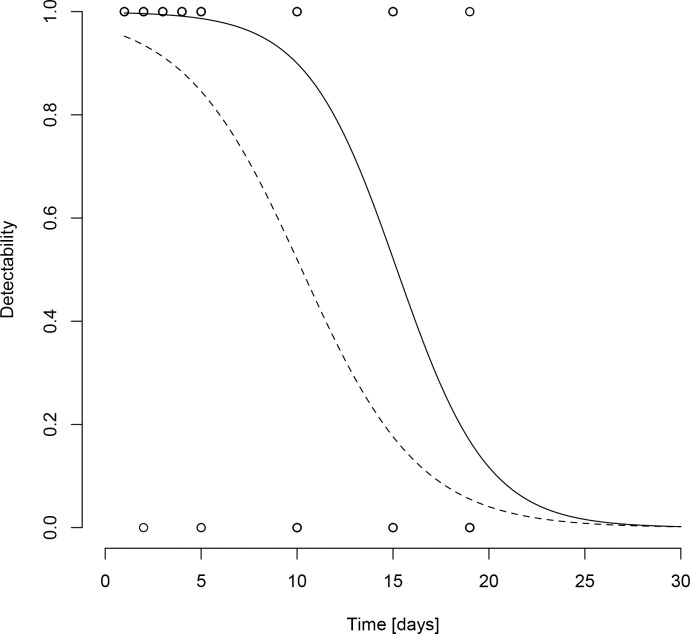
Results from eDNA persistence experiment. *Ae*. *albopictus* eDNA detectability in water as a function of time after the removal of eDNA source material. Circles are data points. Logistic regressions are given for initial densities of 15 (solid line) and 10 (dashed line) larvae per container.

Detectability of eDNA was best described by a model including larval density and days as fixed effects as well as containers as random variable. It significantly depended on the number of days after removal of live material (p < 0.001), as well as on the initial larval density (p = 0.013). DNA was detectable up to 25 days after live material was removed for both densities (Fig 3).

## Discussion

In the present study we developed, validated and compared two different methods (species-specific qPCR and DNA metabarcoding) to identify three invasive mosquito species (*Aedes albopictus*, *Ae*. *j*. *japonicus* and *Ae*. *koreicus*), based on the detection of eDNA from field-collected water samples. Although molecular methods have already been used to identify IMS from tissue specimens [14, 30, 34, 35], this has been achieved here for the first time by analyzing eDNA from water samples. We demonstrated the reliability and efficiency of these eDNA-based approaches, which should establish them as a valuable tool for the early detection and surveillance of invasive mosquito vectors, an issue which presents public health concerns and becomes an emerging problem in Europe and worldwide [59].

Both approaches resulted in reliable and congruent results. Detection probabilities are in the range of other eDNA studies in comparable freshwater systems. For instance, Thomsen *et al*. [60] reported a 82–100% detection probability for various vertebrate and invertebrate taxa (including the dragonfly *Leucorrhinia pectoralis*). Biggs *et al*. [61] found a 99.3% detection probability for the newt *Triturus cristatus* (in ponds surveyed on four occasions during the breeding season). Goldberg *et al*. [62] reported a detection probability of 83–100% for a frog and a salamander species in filtered water samples from five streams. Several causes have been highlighted to explain failure in detecting eDNA from a species which is present in the studied environment (false negatives; [63]), either methodological (e.g. insufficient sensitivity of the method, inappropriate sampling strategy), or related to low DNA quality and/or concentration in the environment (depending on habitat, ecology, density and type of target organisms). Some of these factors may explain the false negatives obtained with the qPCR (N = 3) or the NGS assays (N = 6) in samples found positive with traditional methods, mostly attributable to low eDNA concentration for qPCR and/or unbalanced proportion of target vs. total eDNA present in the water sample for NGS. First, eDNA might have been below the LOD for samples DE4 (a single larvae observed in a relatively large water body) and ES2 (one single qPCR replicate positive out of 12). Even though a reaction can be partially or completely inhibited e.g. by co-extracted chemical inhibitors [50, 64], inhibition tests were negative for these samples. Non-detections due to low specimen numbers have been reported from other studies as well [61, 65], and a positive correlation between density and eDNA detection has been established for various organisms [66, 67]. The negative result for sample IT8 could be due to failure to catch present eDNA when only taking small water volumes or method failure. Second, the 6 samples positive with qPCR but negative with NGS (BA2, CH6, CH10, IT3, IT5 and IT6; S1 Table) are characterized by the presence of highly abundant reads of non-target taxa, which could result in masking low-abundance sequences of the expected target IMS (S3 Table). An inadequate sequencing depth can be one of the causes of missed species [68, 69]. Increasing sequencing depth and the use of blocking primers for abundant non-target species [70] can be a solution to overcome this issue [52]. Interestingly, 4 of these NGS-undetected samples were further analyzed with a deeper sequencing depth (approx. 1 x 10^6^) in an additional NGS run, and 3 of them (BA2, CH6 and IT6) became positive (data not shown).

High degradation of eDNA may be another reason for non-detections. Strickler *et al*. [71] examined the influence of abiotic factors and reported higher degradation rates in water bodies with higher temperatures, more UV-B radiation levels and a not alkaline *pH*. Given that breeding of the studied mosquito species takes place in the warm season and that the water bodies are often very small, rapid degradation may occur.

In the case of the 4 samples which resulted positive for an IMS with qPCR or NGS despite no visual observation during the sampling (CH16, DE5, FR7 and IT10; S1 Table), potential sources of discrepancy can stem from contamination in the laboratory and cross-contamination during qPCR (false positives; [63]), from difficulties to observe cryptic organisms and life stages in the field [72] and/or from DNA persistence in the environment. Since we can exclude the first two causes due to specificity of qPCR primers tested in the laboratory and use of negative controls in the field and during the entire laboratory procedure, failed observation with traditional survey and/or DNA persistence after the target species has left are the most likely factors explaining these results. Several studies found that the decay of DNA in water is rapid and that persistence ranges from days to weeks depending on organisms density [60, 71, 73]. Although performed in controlled laboratory conditions and without the influence of biotic or abiotic environmental factors [74, 75], our results are in line with these studies. Mosquito DNA could still be detected 25 days after the source removal, therefore confirming the recent presence of the target species. In addition, the geographical locations of the water bodies where these four samples have been collected belong to the potential distribution ranges of the detected IMS. These results stress the usefulness of an eDNA approach to detect IMS without prior assessment with traditional monitoring methods. Hence in Switzerland, we found *Ae*. *koreicus* (CH6-CH9) where only *Ae*. *albopictus* was known so far, suggesting that this species is more widespread than expected [17]. Similarly, the range expansion of *Ae*. *j*. *japonicus* has been documented over the past years [23] and we detected this species in Switzerland in a region where its presence has never been documented so far (CH15-19; [46]).

The eDNA approach has clear advantages compared to traditional surveys, including higher detection probability, accurate taxonomic identification and non-invasiveness (reviewed in [76]). Considering our specific case, traditional morphological identification of *Aedes* spp. depends mostly on taxonomic expertise, which is a declining skill [38]. It is generally possible to distinguish *Aedes* adults based on coloration patterns on legs, abdomen and thorax [45]. However, damaged specimens due to the trapping or phenotypic plasticity may complicate morphological determination [30]. Furthermore, not all life stages are equally identifiable by morphological criteria. While it is possible to distinguish *Aedes* larvae (fourth larval stage) in the laboratory, both pupae and eggs are difficult to determine morphologically to the species level [45]. A common approach is to hatch eggs in the laboratory, or rear larvae and pupae to adult stage, which is often unsuccessful, time-consuming and still requires a good expertise for identification [31]. Our study shows that detection probabilities with eDNA-based techniques are in the vast majority of cases higher than those obtained with traditional survey methods based on larval sampling (Tables 1 and 2). This is especially noteworthy given that we used a conservative threshold of 2 replicates for a sample to be regarded as positive in the qPCR assay. Consequently, several occurrences of single positive qPCR replicates (S1 Table) were rejected for the estimation of detection probabilities, contrarily to what prevails in eDNA studies [76]. Considering these samples would result in a higher detection probability for molecular assays and a lower detection probability for the traditional method. Several studies compare eDNA techniques to traditional monitoring such as electrofishing [11, 77] and auditory and visual survey techniques for amphibians [41, 61]. The results indicate that DNA metabarcoding of fish and amphibians provides for equal or better detection than a broad array of traditional methods [52, 78]. In addition to being a very sensitive detection tool, the sampling effort for eDNA techniques is reduced [52] and standardised within studies, hence diminishing human errors influencing detection probabilities [38]. One of the advantages of eDNA techniques is that cryptic life stages may become detectable, rendering the method applicable for longer time periods [41]. Moreover, the risk of an accidental dispersal of alien invasive species or pathogens due to specimen manipulation is minimized, compared with the rearing of mosquito larvae to adults in the laboratory [52]. In contrast, certain information cannot be provided by eDNA analyses, e.g. a differentiation between dead and alive organisms or between different life stages, or quantitative assessments [36, 56, 76], which is admittedly dispensable for the surveillance of IMS but needed for e.g. studies on population dynamics.

We show that detection probabilities with qPCR are in the same range or higher than with NGS (Table 2). Advantages of real-time qPCR over NGS for early and rapid detection of IMS are that no additional steps are needed after the amplification process, and that it is still more cost-effective at least for small sample numbers (e.g. when monitoring new areas for prospective detection of invasive species). However, NGS is a very fast-evolving field [79], and it may be the method of choice in the future as becoming progressively less dependent on the multiplexing of large sample numbers to be cost-effective. As shown by our results, the number of sequence reads per sample is an important parameter in NGS, as target species otherwise detected with qPCR can be missed in some particular circumstances, and this factor (as well as the different number of PCR replicates between qPCR and NGS) could partly explain the differences in detection probability between the two approaches. Importantly, metabarcoding data allow for a more flexible approach (depending on the versatility of the primers employed), without requiring any a priori knowledge of the species potentially detectable in the environmental sample. Consequently, NGS enables the parallel detection of several IMS but also of other (invasive) taxa of interest in a single analysis run, without the constraint to develop species-specific tools and perform several independent analyses. In this context, database coverage for the used molecular marker is a key factor, in particular when dealing with rare and poorly studied newly invasive species, as e.g. *Ae*. *koreicus* in our specific case. The choice of the most suitable method will therefore depend on a study‘s design, the number of samples, the time frame and financial constraints, as well as the questions addressed.

In this study we tested and applied for the first time an environmental DNA approach for the detection of three IMS, based on water samples collected in the field. We demonstrated that there are clear advantages of alternative eDNA-based techniques over traditional surveys for the early detection and surveillance of IMS. Nevertheless, this approach should not generally replace the latter but rather be used in a complementary way [36, 37, 52, 56]. Therefore, we propose the implementation of large-scale surveillance by eDNA sampling including several water bodies with unknown status as a prior step, followed by traditional methods in specific cases of positive findings or when particular data are needed (e.g. information about development stage, densities, etc.). Finally, the ease of water sampling procedures in the eDNA approach tested here, without the need of specific skills, should provide the opportunity to develop citizen science programs for large-scale and effective IMS monitoring and surveillance programs.

## Supporting Information

S1 Fig Results of the *in silico* validation of the Culicidae primers. The analysis was based on the results of an electronic PCR using ecoPCR software [48] on the EMBL-Bank release 127 (April 2016), allowing a maximum of three mismatches per primer. A) sequence logos of the forward and reverse primers illustrating the quality of the match between the primer and its target sequence within the Culicidae taxonomic group; (B) length distribution of the amplified Culicidae sequences (excluding primers); and combined mismatch analysis of the Culicidae-f and Culicidae-r primers (C) for the target group and (D) for all available DNA sequences from the EMBL database (Culicidae in green, non-Culicidae in red).
(TIF)

S2 Fig Standard curves for the qPCR for *Ae*. *albopictus*, *Ae*. *j*. *japonicus* and *Ae*. *koreicus*. The mean cycle threshold (Ct: the cycle at which fluorescence from amplification exceeds the background fluorescence) for 10-fold serial dilutions plotted against the quantity of DNA [log10; 0 = 1 fg x μL^-1^]. Coefficients of correlation (R^2^) were > 99.5% for all tests.
(TIF)

S1 Table Details on sampled sites. (DOC)

S2 Table Origin of target and non-target specimens used for preliminary setup and cross-amplification tests. (DOC)

S3 Table Number of Culicidae DNA sequences obtained by NGS before and after the filtering procedure for each sampled site, and their assignation to taxa after comparison with the local database. (XLS)

S4 Table MIQE Guidelines Checklist. (XLS)
