# Supplementary figures and images for: Detection of Invasive Mosquito Vectors Using Environmental DNA (eDNA) from Water Samples
Source: PLoS One. 2016 Sep 14;11(9):e0162493. doi: 10.1371/journal.pone.0162493 (PMC5023106; doi:10.1371/journal.pone.0162493)

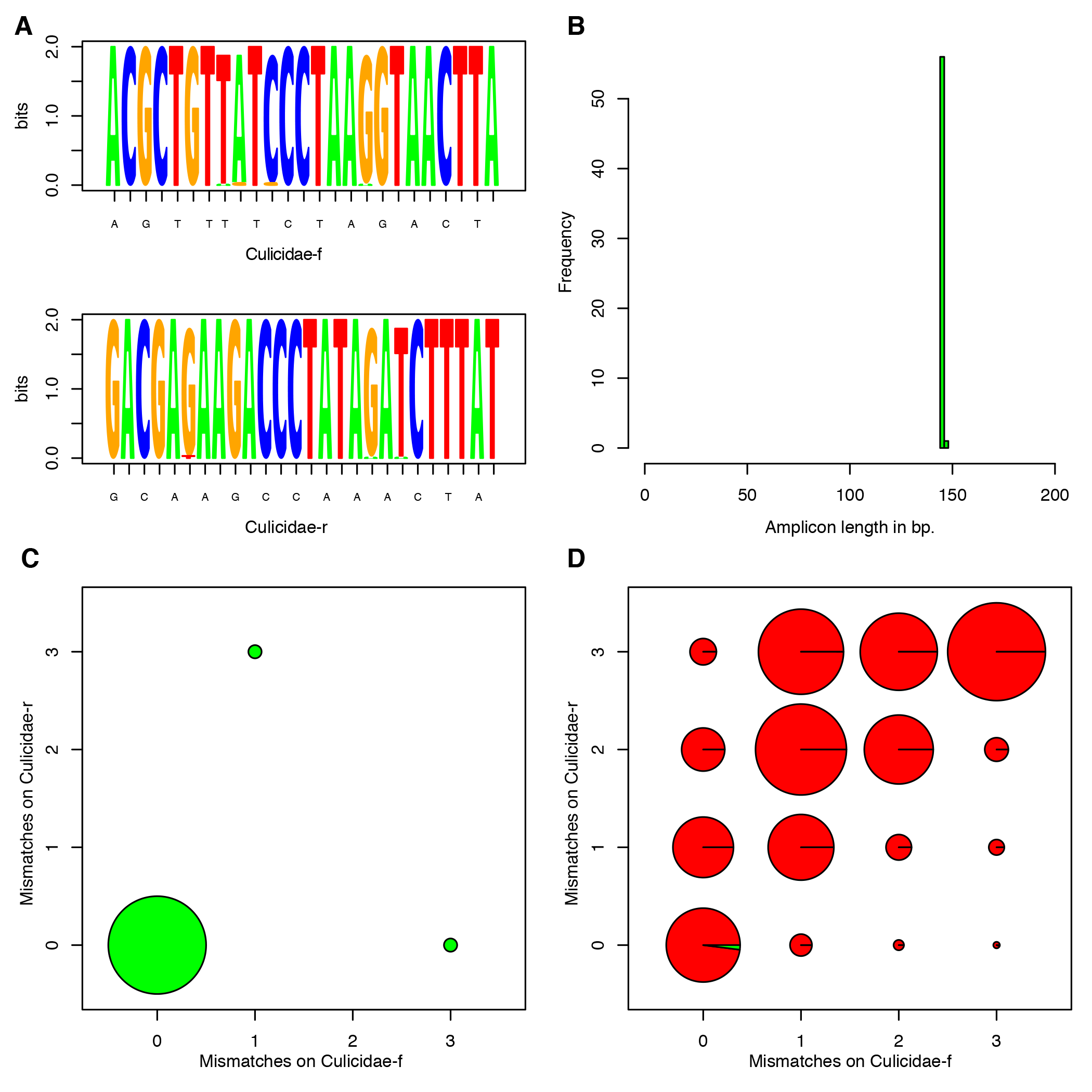

Supplement: S1 Fig — The analysis was based on the results of an electronic PCR using ecoPCR software [48] on the EMBL-Bank release 127 (April 2016), allowing a maximum of three mismatches per primer. A) sequence logos of the forward and reverse primers illustrating the quality of the match between the primer and its target sequence within the Culicidae taxonomic group; (B) length distribution of the amplified Culicidae sequences (excluding primers); and combined mismatch analysis of the Culicidae-f and Culicidae-r primers (C) for the target group and (D) for all available DNA sequences from the EMBL database (Culicidae in green, non-Culicidae in red). (TIF) [file pone.0162493.s001.tif]

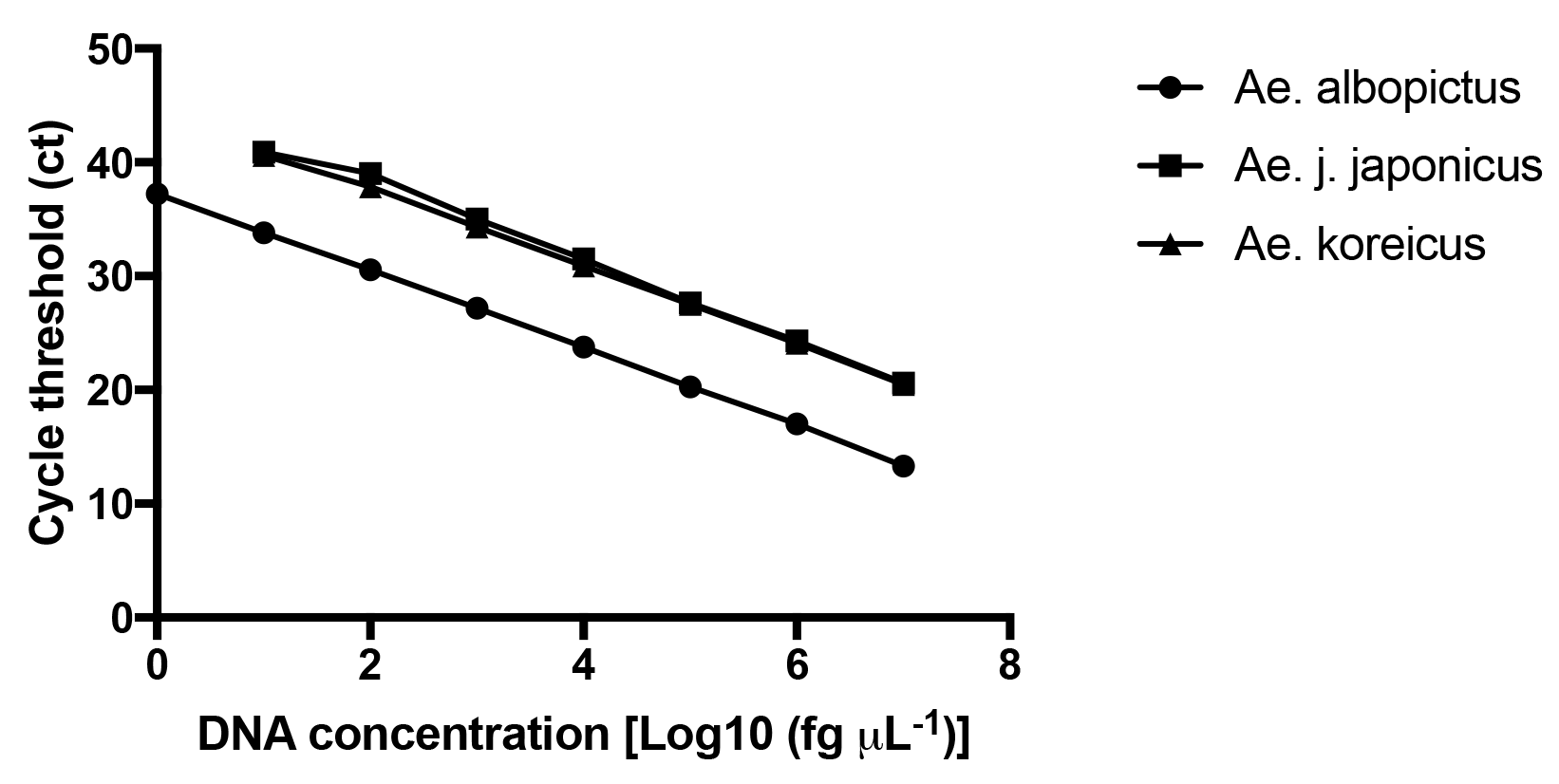

Supplement: S2 Fig — The mean cycle threshold (Ct: the cycle at which fluorescence from amplification exceeds the background fluorescence) for 10-fold serial dilutions plotted against the quantity of DNA [log10; 0 = 1 fg x μL-1]. Coefficients of correlation (R2) were > 99.5% for all tests. (TIF) [file pone.0162493.s002.tif]
