# Supplementary material for: Detection of Invasive Mosquito Vectors Using Environmental DNA (eDNA) from Water Samples
Source: PLoS One. 2016 Sep 14;11(9):e0162493. doi: 10.1371/journal.pone.0162493 (PMC5023106; doi:10.1371/journal.pone.0162493)
Supplement: S1 Table — (DOC) [file pone.0162493.s003.doc]

| **Sample ID** | **Country, Location**  **Coordinates (Lat°, Long °)**  **Type of container**  **Sampler, Date** | **Distribution range (ECDC 2016)**  **Species observed/not observed (+/-) during sampling**  **qPCR (nr. of positives out of 12 replicates); n.t.: not tested**  **NGS (nr. of sequences after filtering)** | | |
| --- | --- | --- | --- | --- |
|  |  | ***Ae. albopictus*** | ***Ae. koreicus*** | ***Ae. j. japonicus*** |
| **AL1** | Albania, Shkodër | yes | no | no |
|  | 42.055554, 19.508060 | - | - | - |
|  | Can | 0 | n.t. | n.t. |
|  | O. Glaizot, 10/06/2015 | 0 | 0 | 0 |
| **AL2** | Albania, Shkodër | yes | no | no |
|  | 42.054813, 19.506086 | - | - | - |
|  | Pond with amphibians | 0 | n.t. | n.t. |
|  | O. Glaizot, 10/06/2015 | 0 | 0 | 0 |
| **BA1** | Bosnia-Herzegovina,  Hutovo | yes | no | no |
|  | 43.6049056, 18.3474361, | + | - | - |
|  | Used tire | 12 | n.t. | n.t. |
|  | F. Schaffner, 12/06/2015 | 59 | 0 | 0 |
| **BA2** | Bosnia-Herzegovina,  Hutovo | yes | no | no |
|  | 43.6029750, 18.3531139 | + | - | - |
|  | Used tire | 12 | n.t. | n.t. |
|  | F. Schaffner, 12/06/2015 | 0 | 0 | 0 |
| **BA3** | Bosnia-Herzegovina,  Neum | yes | no | no |
|  | 43.5417889, 18.0404806 | + | - | - |
|  | Plastic bucket | 12 | n.t. | n.t. |
|  | F. Schaffner, 12/06/2015 | 2,597 | 0 | 0 |
| **CH1** | Switzerland, Zurich | no | no | yes |
|  | 47.400078, 8.552063 | - | - | + |
|  | Stone basin | n.t. | n.t. | 12 |
|  | S. Wagner, J. Schneider, 29/06/2015 | 0 | 0 | 49,248 |
| **CH2** | Switzerland, Zurich | no | no | yes |
|  | 47.402503, 8.574829 | - | - | + |
|  | Plastic cemetery vase | n.t. | n.t. | 12 |
|  | S. Wagner, J. Schneider, 29/06/2015 | 0 | 0 | 57,822 |
| **CH3** | Switzerland, Zurich | no | no | yes |
|  | 47.402242, 8.575752 | - | - | + |
|  | Plastic cemetery vase | n.t. | n.t. | 12 |
|  | S. Wagner, J. Schneider, 29/06/2015 | n.t. | n.t. | n.t. |
| **CH4** | Switzerland, Basel | no | no | yes |
|  | 47.563728, 7.642107 | - | - | - |
|  | Plastic cemetery vase | n.t. | n.t. | 1 |
|  | J. Schneider, 24/07/2015 | 0 | 0 | 0 |
| **CH6** | Switzerland, Lugano | yes | yes | yes |
|  | 46.008145, 8.949629 | + | - | - |
|  | Catch basin | 12 | 2 | n.t. |
|  | E. Flacio, 26/06/2015 | 24 | 0 | 0 |
| **CH7** | Switzerland, Neggio | yes | yes | yes |
|  | 45.983075, 8.878286 | + | - | - |
|  | Plastic can | 12 | 6 | n.t. |
|  | E. Flacio, 14/07/2015 | 119,559 | 238 | 0 |
| **CH8** | Switzerland, Neggio | yes | yes | yes |
|  | 45.983176, 8.878115 | + | - | - |
|  | Building material | 12 | 12 | n.t. |
|  | E. Flacio, 14/07/2015 | 9,253 | 40,281 | 0 |
| **CH9** | Switzerland, Neggio | yes | yes | yes |
|  | 45.983327, 8.877964 | + | - | - |
|  | Building material | 12 | 12 | n.t. |
|  | E. Flacio, 14/07/2015 | 343 | 67,572 | 0 |
| **CH10** | Switzerland, Lugano | yes | yes | yes |
|  | 46.022063, 8.967405 | + | - | - |
|  | Iron can | 9 | 0 | n.t. |
|  | E. Flacio, 14/07/2015 | 0 | 0 | 0 |
| **CH11** | Switzerland, Zurich | no | no | yes |
|  | 47.401806, 8.575408 | - | - | + |
|  | Tone cemetary vase | n.t. | n.t. | 12 |
|  | S. Wagner, J. Schneider, 29/06/2015 | n.t. | n.t. | n.t. |
| **CH12** | Switzerland, Zurich | no | no | yes |
|  | 47.401414, 8.573949 | - | - | + |
|  | Plastic watering pot | n.t. | n.t. | 12 |
|  | S. Wagner, J. Schneider, 29/06/2015 | 0 | 0 | 35,633 |
| **CH14** | Switzerland, Lausanne | no | no | no |
|  | 46.520264, 6.600659 | - | - | - |
|  | Tone cemetary vase | 0 | n.t. | 0 |
|  | J. Schneider, 10/08/2015 | 0 | 0 | 0 |
| **CH15** | Switzerland, Choëx | no | no | no |
|  | 46.244448, 6.954497 | - | - | + |
|  | Used tire | 0 | n.t. | 12 |
|  | J. Schneider, 29/08/2015 | 0 | 0 | 4,580 |
| **CH16** | Switzerland, Choëx | no | no | no |
|  | 46.244514, 6.954405 | - | - | - |
|  | Stone basin | n.t. | n.t. | 12 |
|  | J. Schneider, 24/09/2015 | 0 | 0 | 210,686 |
| **CH17** | Switzerland, Choëx | no | no | no |
|  | 46.244448, 6.954497 | - | - | + |
|  | Used tire | n.t. | n.t. | 12 |
|  | J. Schneider, 24/09/2015 | n.t. | n.t. | n.t. |
| **CH18** | Switzerland, Choëx | no | no | no |
|  | 46.241919, 6.968178 | - | - | + |
|  | Plastic cemetery vase | n.t. | n.t. | 12 |
|  | J. Schneider, 24/09/2015 | 0 | 0 | 220,794 |
| **CH19** | Switzerland, Choëx | no | no | no |
|  | 46.243075, 6.962797 | - | - | + |
|  | Stone basin | n.t. | n.t. | 11 |
|  | J. Schneider, 24/09/2015 | 0 | 0 | 88,769 |
| **DE1** | Germany, Cologne | no | no | yes |
|  | 50.939893, 6.918705 | - | - | - |
|  | Stone bowl | n.t. | n.t. | 0 |
|  | J. Schneider, 25/07/2015 | n.t. | n.t. | n.t. |
| **DE2** | Germany, Cologne | no | no | yes |
|  | 50.900956, 6.944140 | - | - | + |
|  | Stone bowl | n.t. | n.t. | 12 |
|  | J. Schneider, 25/07/2015 | 0 | 0 | 5,445 |
| **DE3** | Germany, Bonn | no | no | yes |
|  | 50.752871, 7.069402 | - | - | + |
|  | Plastic cemetery vase | n.t. | n.t. | 6 |
|  | J. Schneider, 25/07/2015 | 0 | 0 | 80 |
| **DE4** | Germany, Bonn | no | no | yes |
|  | 50.715615, 7.100872 | - | - | + |
|  | Stone bowl | n.t. | n.t. | 0 |
|  | J. Schneider, 25/07/2015 | 0 | 0 | 0 |
| **DE5** | Germany, Cologne | no | no | yes |
|  | 50.897484, 6.940656 | - | - | - |
|  | Stone bowl | n.t. | n.t. | 12 |
|  | J. Schneider, 25/07/2015 | 0 | 0 | 14,297 |
| **ES1** | Spain, Molins de Rei | yes | no | no |
|  | 41.251417, 2.1794 | + | - | - |
|  | Ovitrap tap water,  1 week outdoors | 12 | n.t. | n.t. |
|  | R. Eritja, 17/09/2015 | 39,949 | 0 | 0 |
| **ES2** | Spain,  Sant Just Desvern | yes | no | no |
|  | 41.231030, 2.55113 | + | - | - |
|  | Tank with green algae | 1 | n.t. | n.t. |
|  | R. Eritja, 28/09/2015 | n.t. | n.t. | n.t. |
| **ES3** | Spain,  Esplugues de Llobregat | yes | no | no |
|  | 41.231197, 2.43233 | + | - | - |
|  | Ovitrap tap water,  1 week outdoors | 12 | n.t. | n.t. |
|  | R. Eritja, 17/09/2015 | 108,300 | 0 | 0 |
| **ES4** | Spain, National Parc  Coto de Doñana | no | no | no |
|  | 37.000908, -6.2547096 | - | - | - |
|  | Pond | 0 | n.t. | n.t. |
|  | L. Fumagalli, 09/09/2015 | n.t. | n.t. | n.t. |
| **ES5** | Spain, National Parc  Coto de Doñana | no | no | no |
|  | 37.045313, -6.2718554 | - | - | - |
|  | Pond | 0 | n.t. | n.t. |
|  | L. Fumagalli, 09/09/2015 | n.t. | n.t. | n.t. |
| **FR4** | France, Cotignac | yes | no | no |
|  | 43.530442, 6.150498 | - | - | - |
|  | Pond | 1 | n.t. | n.t. |
|  | P. Christe, 31/07/2015 | 0 | 0 | 0 |
| **FR5** | France, Cotignac | yes | no | no |
|  | 43.527393, 6.150627 | + | - | - |
|  | Fountain | 12 | n.t. | n.t. |
|  | P. Christe, 31/07/2015 | 36,996 | 0 | 0 |
| **FR6** | France, Ramatuelle | yes | no | no |
|  | 43.1821389, 6.6411667 | - | - | - |
|  | Stone basin | 0 | n.t. | n.t. |
|  | L. Fumagalli, 21/07/2015 | 0 | 0 | 0 |
| **FR7** | France, Ramatuelle | yes | no | no |
|  | 43.12529, 6.36399 | - | - | - |
|  | Fountain | 6 | n.t. | n.t. |
|  | L. Fumagalli, 22/07/2015 | n.t. | n.t. | n.t. |
| **FR8** | France, Ramatuelle | yes | no | no |
|  | 43.2146944, 6.6110833 | - | - | - |
|  | Pond | 1 | n.t. | n.t. |
|  | L. Fumagalli, 22/07/2015 | n.t. | n.t. | n.t. |
| **IT2** | Italy, Brugine | yes | no | no |
|  | 45.320883, 11.980873 | + | - | - |
|  | Catch basin | 12 | 0 | n.t. |
|  | F. Montarsi, 28/07/2015 | 95,507 | 0 | 0 |
| **IT3** | Italy, Belluno | yes | yes | no |
|  | 46.14644718, 12.2100715 | + | + | - |
|  | Plastic bucket | 12 | 12 | n.t. |
|  | F. Montarsi, 17/07/2015 | 0 | 30 | 0 |
| **IT4** | Italy, Belluno | yes | yes | no |
|  | 46.156055, 12.2233056 | - | + | - |
|  | Flowering pot | 0 | 2 | n.t. |
|  | F. Montarsi, 17/07/2015 | 0 | 114 | 0 |
| **IT5** | Italy, Belluno | yes | yes | no |
|  | 46.141761, 12.183285 | - | + | - |
|  | Stone bowl | 0 | 2 | n.t. |
|  | F. Montarsi, 17/07/2015 | 0 | 0 | 0 |
| **IT6** | Italy, Asolo | yes | yes | no |
|  | 45.810446, 11.891293 | + | + | - |
|  | Tank | 12 | 12 | n.t. |
|  | F. Montarsi, 18/07/2015 | 93,779 | 0 | 0 |
| **IT7** | Italy, Rivamonte Agordino | yes | yes | no |
|  | 46.257628, 12.042805 | - | - | - |
|  | Metal container | 0 | 0 | n.t. |
|  | F. Montarsi, 17/07/2015 | n.t. | n.t. | n.t. |
| **IT8** | Italy, Rivamonte Agordino | yes | yes | no |
|  | 46.270184, 12.035322 | - | + | - |
|  | Metal rain barrel | 0 | 0 | n.t. |
|  | F. Montarsi, 17/07/2015 | n.t. | n.t. | n.t. |
| **IT9** | Italy, Volterra | yes | no | no |
|  | 43.400667, 10.861144 | - | - | - |
|  | Stone bowl | 0 | 0 | n.t. |
|  | J. Schneider, 07/08/2015 | n.t. | n.t. | n.t. |
| **IT10** | Italy, Bibbona | yes | no | no |
|  | 43.287375, 10.582842 | - | - | - |
|  | Tree hole | 12 | 0 | n.t. |
|  | J. Schneider, 06/08/2015 | 147,784 | 0 | 0 |
