# Supplementary material for: Detection of Invasive Mosquito Vectors Using Environmental DNA (eDNA) from Water Samples
Source: PLoS One. 2016 Sep 14;11(9):e0162493. doi: 10.1371/journal.pone.0162493 (PMC5023106; doi:10.1371/journal.pone.0162493)
Supplement: S2 Table — (DOC) [file pone.0162493.s004.doc]

**Table S2** Origin of target and non-target mosquito specimens used for preliminary set up and cross-amplification tests.

| **Target mosquito species** | | |
| --- | --- | --- |
| **Species** | **Life stage** | **Origin** |
| *Aedes albopictus* | 5 adults, 5 larvae | 1, sampled in France |
|  | 2 adults | 2, sampled in Treviso province (IT) |
| *Ae. j. japonicus* | 8 adults | 3, laboratory colony origin USA |
| *Ae. koreicus* | 5 adults, 2 larvae | 2, laboratory colony |
| **Non-target mosquito species** | | |
| **Species** | **Life stage** | **Origin** |
| *Ae. cinereus/geminus* | 1 female | 1, sampled in Switzerland |
| *Ae. vexans* | 1 adult | 1, sampled in Switzerland |
| *Anopheles plumbeus* | 1 adult | 1, sampled in Switzerland |
| *Culex hortensis* | 1 larvae | 4, sampled in Switzerland |
| *Culex pipiens* | 1 larvae | 4, sampled in Switzerland |
| *Culex territans* | 1 larvae | 1, sampled in Switzerland |
| *Culiseta alaskaensis* | 1 female | 1, sampled in Switzerland |
| *Culiseta annulata* | 1 larvae | 1, sampled in Switzerland |
| *Culiseta longiareolata* | 1 larvae  1 female | 3, sampled in Switzerland |
| *Culiseta morsitans* | 1 larvae | 1, sampled in Switzerland |
| *Ochlerotatus annulipes* | 1 male | 1, sampled in Switzerland |
| *Ochlerotatus geniculatus* | 1 larvae | 1, sampled in Switzerland |
| *Ochlerotatus rusticus* | 1 larvae | 1, sampled in Switzerland |
| *Orthopodomia pulcipalpis* | 1 male | 1, sampled in Switzerland |

1: O. Glaizot, Museum of Zoology, Lausanne, Switzerland

2: F. Montarsi, Istituto Zooprofilattico Sperimentale delle Venezie, Italy

3: A. Mathis, University of Zurich, Switzerland

4: J. Delhaye, University of Lausanne, Switzerland
